# Generalizations of the Jaccard index and Sørensen index for assessing agreement across multiple readers in object detection and instance segmentation in biomedical imaging

**DOI:** 10.1117/1.JMI.10.6.065503

**Published:** 2023-12-14

**Authors:** Madeleine S. Durkee, Kyle Lleras, Karen Drukker, Junting Ai, Thao Cao, Gabriel Casella, Deepjyoti Ghosh, Marcus R. Clark, Maryellen L. Giger

**Affiliations:** a University of Chicago, Department of Radiology and Committee on Medical Physics, Chicago, Illinois, United States; b University of Chicago, Section of Rheumatology and Gwen Knapp Center for Lupus and Immunology Research, Department of Medicine, Chicago, Illinois, United States

**Keywords:** reader performance, reader agreement, algorithm performance, supervised learning, biomedical imaging

## Abstract

**Significance:**

Manual annotations are necessary for training supervised learning algorithms for object detection and instance segmentation. These manual annotations are difficult to acquire, noisy, and inconsistent across readers.

**Aim:**

The goal of this work is to describe and demonstrate multireader generalizations of the Jaccard and Sørensen indices for object detection and instance segmentation.

**Approach:**

The multireader Jaccard and Sørensen indices are described in terms of “calls,” “objects,” and number of readers. These generalizations reduce to the equations defined by confusion matrix variables in the two-reader case. In a test set of 50 cell microscopy images, we use these generalizations to assess reader variability and compare the performance of an object detection network (Yolov5) and an instance segmentation algorithm (Cellpose2.0) with a group of five human readers using the Mann–Whitney U-test with Bonferroni correction for multiplicity.

**Results:**

The multireader generalizations were statistically different from the mean of pairwise comparisons of readers (p<0.0001). Further, these multireader generalizations informed when a reader was performing differently than the group. Finally, these generalizations show that Yolov5 and Cellpose2.0 performed similarly to the pool of human readers. The lower bound of the one-sided 90% confidence interval for the difference in the multireader Jaccard index between the pool of human readers and the pool of human readers plus an algorithm were −0.019 and −0.016 for Yolov5 and Cellpose2.0, respectively.

**Conclusions:**

Multireader generalizations of the Jaccard and Sørensen indices provide metrics for characterizing the agreement of an arbitrary number of readers on object detection and instance segmentation tasks.

## 1 Introduction

The “ole” machine learning adage of “garbage in, garbage out” highlights the necessity of clean and reliable training data with high-quality, consistent annotations. Manual annotation of data to serve as the reference standard, or “ground truth,” is necessary for training supervised learning algorithms to perform a variety of tasks including image-based tasks, such as image classification, segmentation, object detection, and instance segmentation.^1^^–^^3^ Manual annotations of images are often treated as the gold standard and are used as references in computing cost functions and measuring algorithm performance. Deep convolutional neural networks (DCNNs) continue to demonstrate high accuracy for several computer vision tasks, including object detection and instance segmentation tasks.^4^^–^^6^ As many of these algorithms are fully supervised learning algorithms, their performance directly depends on the quality and reliability of human-defined ground truth. Ambiguities and inconsistencies in the ground-truth training data will result in less accurate and less consistent performance by a DCNN.^7^^,^^8^

In biomedical image annotation, it is well established that manual annotations, which are subjective, can be noisy and inconsistent.^9^^,^^10^ Radiologists and pathologists can have varying opinions on image classification tasks, both compared with other experts (interreader variability) and compared with themselves at a different time point or in different reading conditions (intrareader variability).^11^^–^^14^ Human readers, even those trained extensively in discerning slight abnormalities in medical images, are seriously challenged by object counting and object detection in images.^15^^,^^16^ This also extends to instance segmentation, which is the generation of semantic segmentations for each detected object. Because of this known variability and the inconsistency in human readers defining ground truth in images, it is valuable to quantify the extent to which readers agree on a specific task.^10^ This quantitative assessment can better inform researchers on the quality of the annotated training data for training supervised learning algorithms and provide a more realistic benchmark for algorithm comparison. For some computer vision tasks, quantitative measures of multireader variance are well developed. Multireader multicase (MRMC) receiver operating curve (ROC) analysis is designed to optimize the acquisition of manual annotations from multiple readers for image classification tasks.^17^^,^^18^ To the best of our knowledge, no equivalent methods exist for improving object level segmentations, in which the noise in the image annotations is compounded by within-image variability in the number of objects called and variations in each individual object mask. For many object detection tasks, no absolute reference standard exists outside of human annotations, which are known to be noisy. Because of this lack of a reference standard, MRMC analysis becomes intractable. Additionally, true negative (TN) objects are undefined for object detection tasks, which prevents ROC analysis from being a useful evaluation tool. Human-in-the-loop (HITL) training methods allow the reader to work with an algorithm to quickly train object detection and instance segmentation models.^19^^,^^20^ In HITL methods, a human reader manually corrects the output of an algorithm to generate more training data for fine-tuning that same model. Such training schemes reduce the time and resource load of generating training datasets. However, manual annotation of test sets without computer assistance is still necessary to quantify the performance of HITL-trained object detection models. As discussed, these manual annotations are an imperfect standard. Some would argue that the performance of a human annotating with the aid of a computer is a better reference standard than the human annotating alone, but unfortunately—with no perfect standard to compare to—there is no quantitative solution to answer this philosophical disagreement.

Existing metrics for assessing reader variability in object detection tasks rely on pairwise comparisons of readers.^21^ The most commonly used object detection metric, mean average precision, is the area under the precision–recall curve. Both precision and recall are calculated from the confusion matrix variables of true positives (TPs), false positives (FPs), and false negatives (FNs). Note that TNs are undefined for all object detection tasks, prohibiting the calculation of specificity. Importantly, all of these confusion matrix variables are defined through pairwise comparisons of a single reader compared with an established ground truth of two readers but not for the multireader case.^21^ Free-response receiver operating characteristic analysis handles object detection and localization tasks well but is not designed for the multireader case.^22^ The distribution of pairwise comparisons of readers paints an incomplete picture of the multireader agreement across a dataset as the datapoints in a distribution of pairwise comparisons are inherently nonindependent. Because of this lack of independence, comparing a nonhuman reader, i.e., a computer algorithm, with a collection of human readers becomes problematic within this approach. One could average all pairwise comparisons per image, but the disagreement between one pair of readers might not be accurately reflected. Additionally, defining the degree of consensus across readers for object detection is a nontrivial task.^23^ The simultaneous truth and performance level estimation algorithm^24^ generates consensus segmentations across multiple readers to define ground truth. However, this algorithm acts on a binary mask and therefore discards all object-level information when defining consensus. In cases in which objects are spatially distinct and nonoverlapping, the consensus segmentations can easily be separated back into discrete objects. However, in the case of cell detection and segmentation, cells can be packed very tightly with spatially adjacent boundaries.^25^ Additionally, some readers might define two to three objects within a single object called by another reader.

We present two metrics to assess the agreement of an arbitrary number of readers on object detection and instance segmentation tasks. Each can be used to directly assess whether an object detection algorithm or instance segmentation algorithm falls within the distribution of human readers. We also discuss methods for defining reader consensus while maintaining object-level information, as the calculation of the multireader generalizations that we discuss requires the definition of discrete objects. We demonstrate the functionality of these metrics by evaluating reader agreement across five human readers in the task of cell instance segmentation. We also compare two DCNNs with the group of human readers.

## 2 Methods

Here we describe generalized forms of the Jaccard and Sørensen indices that can be applied when evaluating agreement across an arbitrary number of readers. Specifically, we discuss these metrics with respect to object detection and instance segmentation tasks.

### 2.1 Generalized Metrics for Object Detection and Instance Segmentation

The following sections define generalizations of the Jaccard and Sørensen indices, which can account for more than simple pairwise comparisons of readers. The canonical forms of the Jaccard and Sørensen indices [Eqs. (1) and (2)] are defined with confusion matrix variables (TPs, FPs, TNs, and FNs).

$$\text{Jaccard index}=\frac{\mathrm{TP}}{\mathrm{TP}+\mathrm{FP}+\mathrm{FN}},$$ (1)

$$\text{Sørensen index}=\frac{2\mathrm{TP}}{2\mathrm{TP}+\mathrm{FP}+\mathrm{FN}}.$$ (2)

Note that these equations do not use the TN variable, as TNs are undefined in object detection tasks. Confusion matrix variables inherently require exactly two items to compare. In the multireader generalizations of these metrics, we seek to compare an arbitrary number of readers, so we define the terms “calls” and “agreements” to describe multireader agreement. The term “calls” refers to the number of putative objects called across a given number of readers, and the term “agreements” refers to the total number of objects called after calculating consensus across all readers.

#### 2.1.1 Defining agreement on a single object from multiple calls

**Fig. 1:**
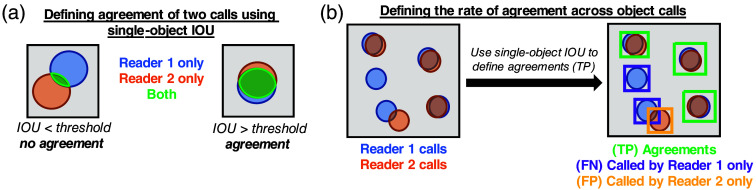
(a) A pixel-level IOU threshold can be used to define overlapping calls from two readers as an agreement or TP. Overlapping pixels are highlighted in green. (b) A toy example of Jaccard and Sørensen indices as defined by confusion matrix variables. In this analogy, reader 1 is defined as the gold standard, and therefore objects called only by reader 1 are defined as FN, purple boxes. Reader 2 is therefore analogous to an algorithm, and therefore objects called only by reader 2 are defined as FP, yellow box. Objects that the two readers agree on are TP, green boxes.

Determining whether two or more readers agree in defining a single object requires a pixel-level assessment of the consensus between readers for that object. Pixel-level metrics, including intersection over union (IOU) and the Dice-Sørensen coefficient (DSC), are used to help define whether two overlapping segmentations are in fact defining the same object. The definition of an agreement (or a TP object) requires the IOU or DSC calculated between two calls to be more than some predefined threshold [Fig. 1(a)]. If the IOU or DSC calculated between two calls falls below that threshold, those two calls are defined as two separate objects rather than one.

Here we would like to note that in terms of confusion matrix variables, the equations for Jaccard index and IOU are equivalent, and Sørensen index and DSC are equivalent. To clearly separate the discussion of pixel-level agreement and object agreement, we use IOU and DSC when referring to the pixel-level agreement used for defining agreement on a single object, and we use the terms Jaccard and Sørensen indices when defining the agreement across multiple objects in an image or dataset.

#### 2.1.2 Multireader generalization of the Jaccard Index

The Jaccard index [Eq. (1)] is a measure of algorithm performance that relates the number of correctly identified objects (TP) to the sum of the correctly identified objects, the erroneous predictions, and the missed objects (TP + FP + FN). The Sørensen index is a similar metric, but with TP more heavily weighted. In comparing two readers, the Jaccard index is equivalent to a ratio of the number of objects that the two readers agree on to the number of agreed upon objects plus the number of objects identified only by each reader [Fig. 1(b)]. The Sørensen index [Eq. (2)] is a similar comparison, yet with agreements more heavily weighted. Note that, when comparing two expert human readers, either reader can be treated as the reference standard; however, FP and FN will flip depending on which reader is chosen. In both the Jaccard and Sørensen indices, FP and FN can be interchanged without affecting the value of the metric. Reader pairs are therefore commutable when evaluating these metrics.

Using this analogy of the number of agreements relative to the total number of calls, we present the multireader generalization of the Jaccard index [Eq. (3)]. As mentioned earlier, the definition of a TP object, also referred to here as an agreement, is dependent on the pixel-level IOU threshold demonstrated in Fig. 1(a). The multireader Jaccard index uses the total number of calls (C) across all readers ($N_{R}$) and the total number of objects (O), which can vary in their level of agreement across readers. Objects therefore have the attribute of being called by k readers, ranging from 1 to the maximum number of calls ($N_{C}$), where $N_{C}=N_{R}$:

$$\text{multireader Jaccard index}\;(\mathrm{IOU})=\frac{\sum_{j=1}^{N_{R}}C_{j}-\sum_{k=1}^{N_{C}}O_{k}(\mathrm{IOU})}{\left(N_{R}-1)\sum_{k=1}^{N_{C}}O_{k}(\mathrm{IOU}\right)}.$$ (3)

In the two-reader case ($N_{R}=2$), the total number of calls ($\sum_{j=1}^{N_{R}}C_{j}$) is represented in terms of confusion matrix variables as: 2TP + FP + FN. Similarly, the total number of objects ($\sum_{k=1}^{N_{C}}O_{k}(\mathrm{IOU})$) is interpreted as: (TP + FP + FN). Using these confusion matrix representations, the multireader generalization simplifies to Eq. (1).

#### 2.1.3 Multireader generalization of the Sørensen Index

The Sørensen index, which is also known as the F1 score, can also be applied to evaluate the algorithm performance or reader agreement for object detection and instance segmentation tasks. This metric more heavily weights TP—or agreements—in its calculation [Eq. (2)]. For pairwise comparisons, the relationship between the Sørensen index and the Jaccard index is Sørensen = 2*Jaccard/(1 + Jaccard). More generally, for an arbitrary number of readers, we state this as Sørensen = $N_{R}*\text{Jaccard}/(N_{R}-1+\text{Jaccard})$, where $N_{R}$ is the number of readers performing a given task. Applying this relationship to Eq. (3), we define the multireader Sørensen index as Eq. (4). Given the two-reader case with confusion matrix representations above, this generalized description simplifies to the Sørensen index defined in Eq. (2) for any given IOU:

$$\text{multireader Sørensen index}\;(\mathrm{IOU})=\frac{N_{R}(\sum_{j=1}^{N_{R}}C_{j}-\sum_{k=1}^{N_{C}}O_{k}(\mathrm{IOU}))}{\left(N_{R}-1{)}^{2}\sum_{k=1}^{N_{C}}O_{k}(\mathrm{IOU})+\sum_{j=1}^{N_{R}}C_{j}-\sum_{k=1}^{N_{C}}O_{k}(\mathrm{IOU}\right)}.$$ (4)

#### 2.1.4 Computing consensus across an arbitrary number of readers

**Fig. 2:**
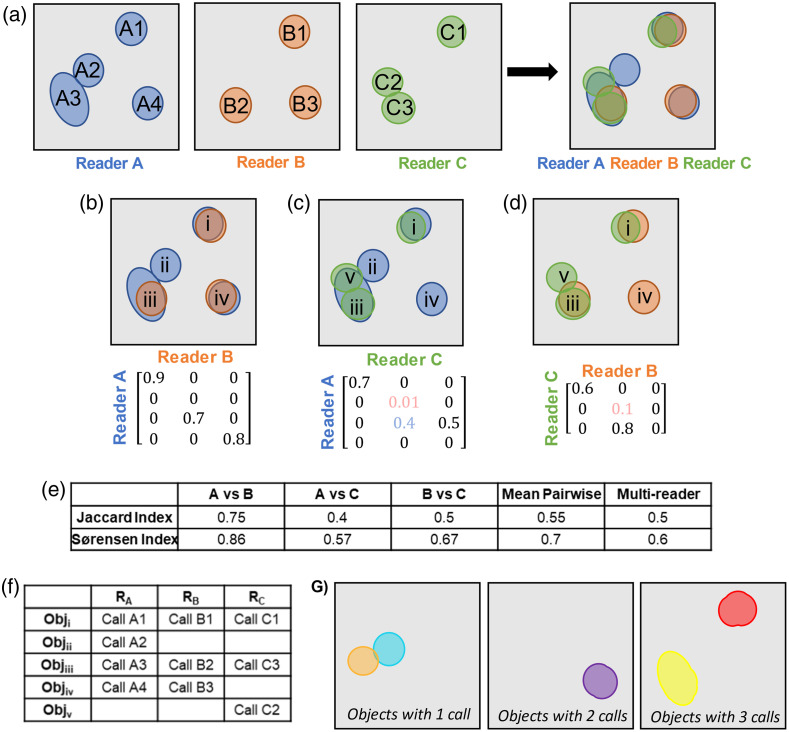
Defining consensus across multiple readers. (a) A toy example of an image segmented by three separate readers: reader A (blue), reader B (orange), and reader C (green). Unique calls from each reader are indicated on each object. (b) A pairwise comparison of readers A and B from (a). The corresponding agreement matrix for these two readers is depicted below the graphic. Numbers shaded in blue would be rejected from NMS, and numbers shaded in pink would be rejected by filtering with an IOU or DSC threshold. (c) A comparison of readers A and C with the corresponding agreement matrix. (d) A comparison of readers B and C with the corresponding agreement matrix. Note that the agreement matrices in (b)–(d) are estimations based on the graphic, not exact calculations. Unique objects in (b)–(d) are indicated with Roman numerals. (e) The pairwise Jaccard and Sørensen indices are shown for (b)–(d) along with the mean pairwise calculation and the multireader generalization. (f) The final match matrix associates each call from each reader ($R_{N}$) with a final object (${Obj}_{M}$). (g) The match matrix can be mapped to a multilayer image representation of reader agreement, with each agreed upon object residing in the layer corresponding to the number of readers who called it.

**Fig. 3:**
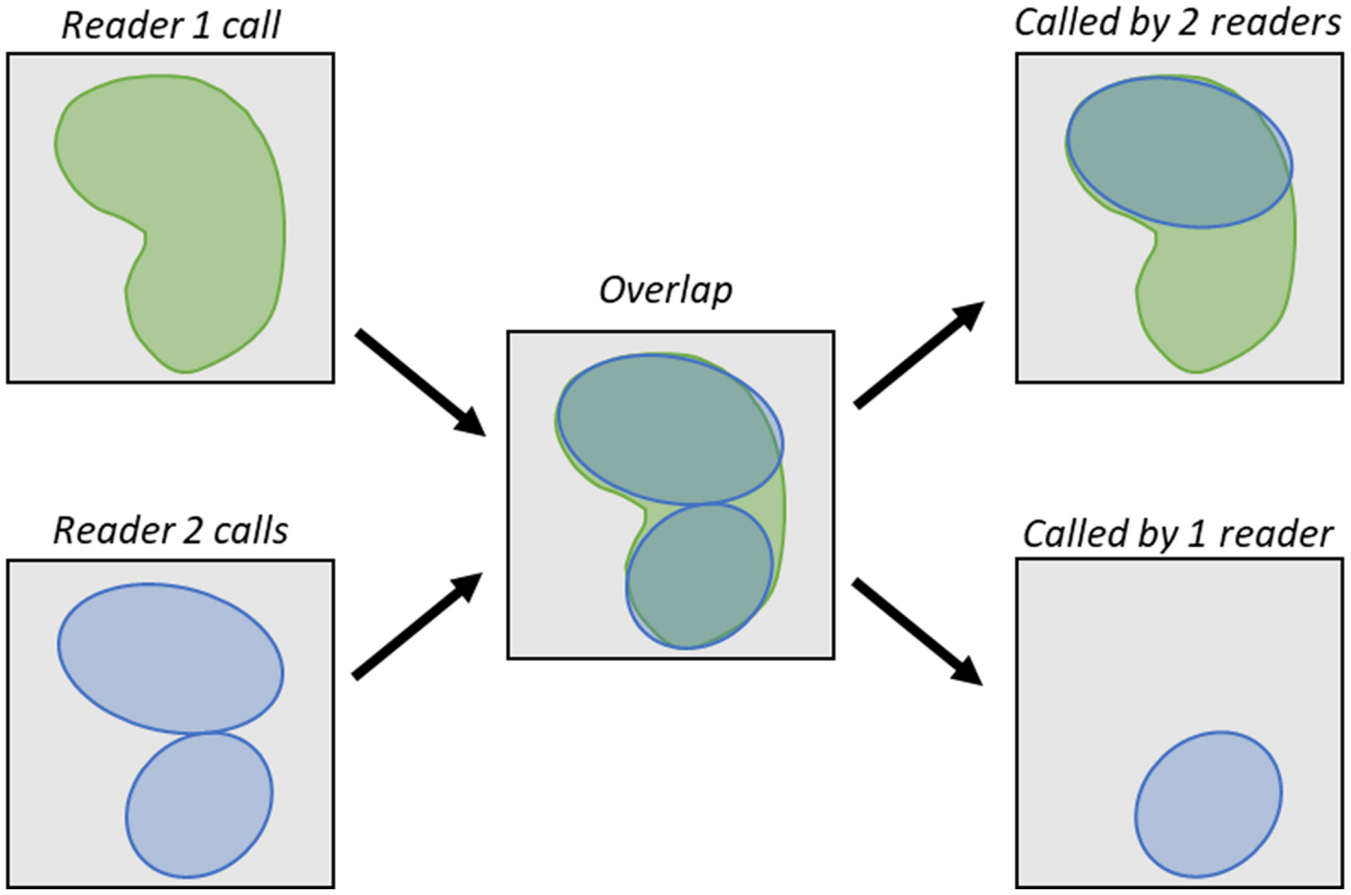
NMS helps to define agreement in cases in which a reader (reader 2, blue) fragments an object called by another reader (reader 1, green). Only the fragment call from reader 2 is accepted as agreeing with the call from reader 1. The second fragment is counted as only being called by one reader, despite having a high overlap with the call from reader1. NMS ensures that all calls are accounted for without counting one reader’s call as agreeing with multiple of another reader’s calls. Note that, although we discuss reader 2 as “fragmenting” reader 1’s call, both readers are equally correct. Reader 1 could also be considered to consolidate unique calls from reader 2.

Defining the number of objects and the degree to which calls from multiple readers agree on an object is not a trivial task. First, an agreement must be determined by a threshold of some pixel-level metric, such as IOU or DSC. A toy example for defining consensus is depicted in Fig. 2. To define consensus, we first look at each pairwise comparison of readers. The number of objects and the number of calls per object can be found with an agreement matrix [Figs. 2(b)–2(d)]. This pairwise agreement matrix is an M×N matrix, where M is the number of calls from reader A and N is the number of calls from reader B. For each pairwise comparison of calls, the value of a given segmentation metric, such as IOU or DSC, is computed. From this matrix, we reject all values less than our predetermined threshold by setting them equal to zero and perform a version of nonmaximum suppression (NMS) (Fig. 3). NMS traditionally rejects overlapping predictions of an object detection algorithm, except for the one with the highest prediction score, therefore “suppressing” predictions for which the algorithm is less “confident.” Here we are comparing readers and have no scores; each reader’s call has equal weight. Therefore, we use a pixel-level overlap metric (IOU or DSC) to reject objects in cases in which multiple calls from one reader intersect with a single call from another reader. Because some readers may call multiple objects where another reader calls a single object (referred to as fragmenting), two calls are only considered an agreement if their pixelwise IOU or DSC is the maximum in all axes of the agreement matrix [Figs. 2(b)–2(d)]. Conceptually, this ensures that only the best match of overlapping calls from reader A and reader B are defined as an agreement.

For an arbitrary number of readers, a separate agreement matrix is computed for all unique comparisons of $N_{R}$ readers, which is $N_{R}(N_{R}-1)/2$ comparisons. After thresholding and NMS across all agreement matrices, we create a match matrix, which index matches every object in our pairwise agreement matrices to every other pairwise comparison of readers for that image. This yields a matrix of $N_{A}$ agreements by $N_{R}$ readers, with each element in this matrix corresponding to the index of the matched call from each reader [Fig. 2(e)]. If a reader did not call an object where other readers did, the element for that reader at that object is left blank. An image-level representation of this match matrix is depicted in Fig. 2(f). This representation contains $N_{R}$ layers of X×Y matrices, where X and Y are the dimensions of the image in question. Each layer k of this representation contains the objects called by k readers. If a single set of ground-truth objects called by k readers is desired, this representation can be used to filter the objects by how many readers called each to define a singular “ground truth” from these segmentations.

### 2.2 Dataset Acquisition of Ground Truth from Multiple Readers

**Fig. 4:**
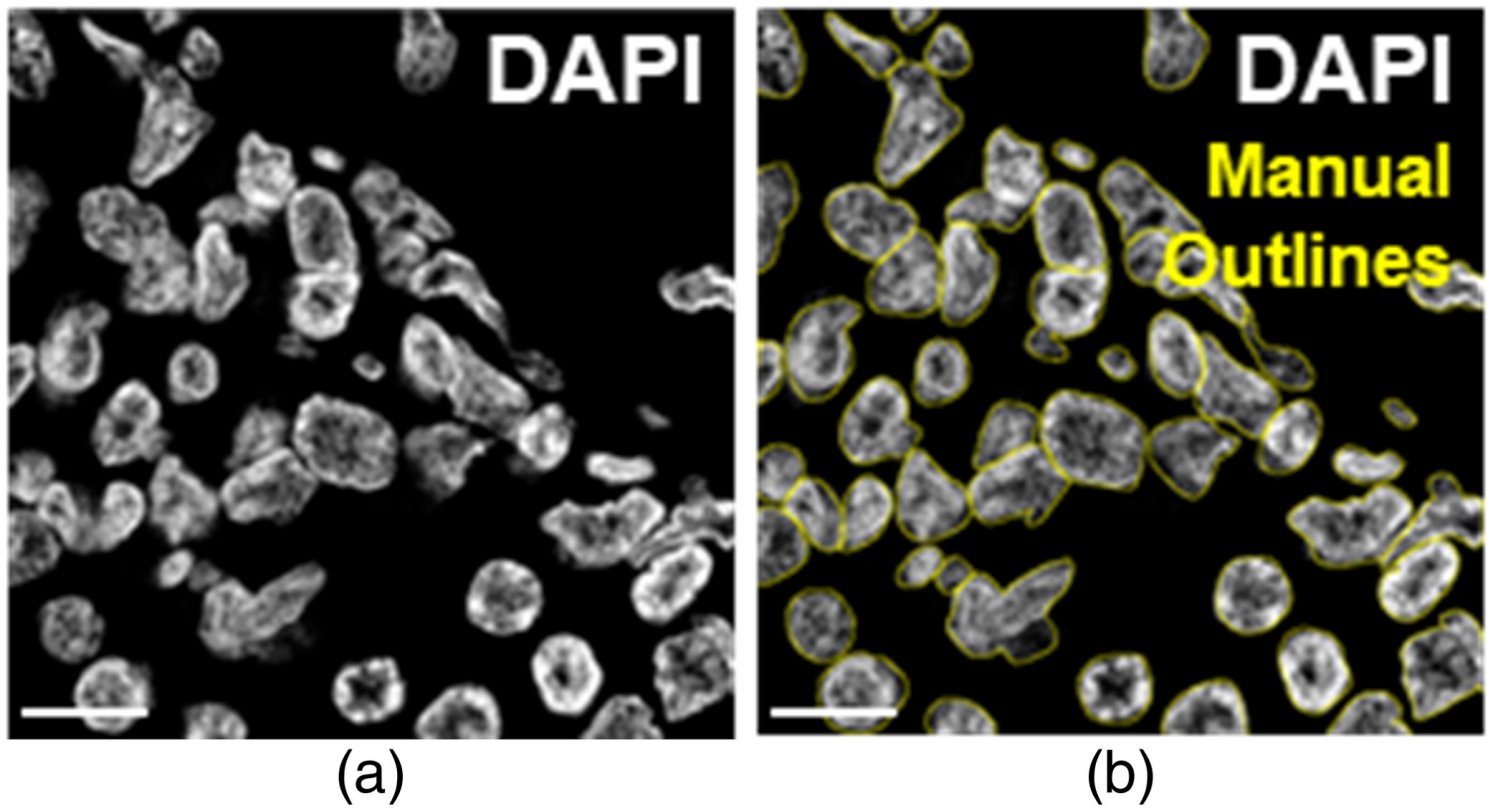
Representative image and manual segmentations. (a) Representative single-channel fluorescence image of DAPI (cell nucleus marker) from the manually annotated dataset. (b) Image with overlay of manual outlines from a single human reader. Scale bar=10  μm.

For this study, we used an existing dataset of fluorescence microscopy images of renal biopsies from patients diagnosed with lupus nephritis. These data were previously obtained from the University of Chicago Human Tissue Resource Center for a separate study.^26^ Briefly, biopsies in this dataset were stained with several immunofluorescence markers and imaged on a Large Format Caliber ID fluorescence confocal microscope at a magnification of 63× with a pixel size of 0.221  μm. For this analysis, we extracted the DAPI (ds-DNA) channel from these multiplex images to identify all nucleated cells [Fig. 4(a)].

### 2.3 Annotations

To demonstrate the utility of the proposed generalized, multireader performance metrics, we acquired annotations on our dataset from both human and computer readers. Five human readers provided manual annotations, and Yolov5 and Cellpose2.0 were used to provide object detection (bounding box) and instance segmentation annotations, respectively.

#### 2.3.1 Human readers

We recruited and trained five readers to perform manual segmentations of cell nuclei in DAPI images to assess reader agreement. Prior to this experiment, readers had minimal to no experience in cellular imaging and segmentation. All readers completed 15 to 20 h of training prior to segmenting images for analysis. All images used for training data were independent of the data used for reader comparisons. When manually segmenting cells, readers were limited to 3-h sessions to minimize fatigue. All readers were provided with touchscreen tablets and styluses. Readers segmented cells by outlining each individual cell in an image with the freehand tool in ImageJ^27^ [Fig. 4(b)]. Readers segmented 50 images patches of 512×512  pixels, which were sampled from whole-section images from five lupus nephritis patients by extracting 10 random and unique areas from each sample. Readers independently segmented images “from scratch” with no assistance from each other or from an algorithm.

#### 2.3.2 Machine learning algorithms (DCNNs)

For a separate study, our group trained a Yolov5^28^ to detect cell nuclei in triple-negative breast cancer (TNBC) images. The network was trained on 512×512-pixel DAPI image tiles extracted from fluorescence confocal images of TNBC biopsies. The training set included 100 image tiles from three TNBC biopsies (∼33 image tiles per biopsy). The validation set included 12 images from a fourth TNBC biopsy. Training was stopped when the performance on the validation set stopped increasing using the early stopping function in Keras.^28^ The trained Yolov5^29^ was deployed on the 50-image lupus nephritis test dataset described above. Not only was this test set from an independent set of patients, but these patient biopsies also originated from a different tissue than the training data (breast versus kidney) and sampled a different pathology (TNBC versus lupus nephritis). Note that Yolov5 outputs object bounding boxes rather than detailed object outlines.

We also deployed Cellpose2.0,^19^ the state-of-the-art instance segmentation algorithm for cellular images, on the test set. We used the built-in “nuclei” model from Cellpose2.0 with no additional fine-tuning.

All computation was accomplished using our Radiomics Analysis Commons for Deep Learning in Biomedical Discovery, on a S10 HPE Superdome Flex computational server, which contains 256 Xeon Gold 6130 CPU cores, 3 TB of DDR4 ECC RAM memory, 24 TB of NVMe SSD storage, and 16 Nvidia Tesla V100 32GB GPU accelerators.

### 2.4 Assessment of Agreement

Here, we describe our analyses for validating the multireader generalizations of the described performance metrics to understand the degree of agreement among human readers and between a group of human readers and two computer readers in performing object detection and instance segmentation tasks.

#### 2.4.1 Interreader agreement

Additionally, agreement was computed for each image by comparing readers in a pairwise fashion using the equations in Fig. 1(a) and across all readers using the multireader generalizations of the Jaccard and Sørensen indices. A putative cell was defined as an agreement by two readers if the IOU between the cell segmentations was greater than a given threshold. Reader agreement was compared across several IOU thresholds to ensure that performance metrics were trending down as the agreement criteria became more stringent.

#### 2.4.2 Intrareader agreement

To assess reader consistency, three readers were asked to resegment the DAPI image patches 1 to 3 months after they first completed manual annotations for the dataset. The level of agreement of readers with themselves was evaluated using the pairwise Jaccard and Sørensen indices. Note that, for two readers (in this case a person and their past self), the multireader generalized metrics are equivalent to the pairwise metrics.

#### 2.4.3 Reader quality

Individual readers were compared with the group of the other readers by evaluating the group agreement without each reader. The multireader Jaccard index was compared between all readers and each unique set of $N_{R}-1$ readers.

#### 2.4.4 Human and computer agreement

The outputs of the Yolo5 and Cellpose2.0 were compared with the five human readers. In both comparisons, we treated the algorithm as a sixth reader and compared its agreement with the original group of five human readers. Performance of Yolov5 and Cellpose2.0 were evaluated relative to the group of human readers using the multireader generalizations of the Jaccard and Sørensen indices. The group of human readers was compared with the group of humans plus the algorithm, and noninferiority testing was used to test whether the group agreement decreased when adding the algorithm to the group. For evaluations involving Yolov5, bounding boxes were computed from the manual annotations by extracting the minimum and maximum x and y coordinates for each object identified by a reader.

Additionally, multiple sets of ground-truth annotations were established from the multireader match matrices. The manual annotations from the five human readers were combined to create five separate ground-truth sets based on how many readers agreed on each cell call. In the first ground-truth set, a segmented object was included if a minimum of one human reader called that object. In the second set, only segmented objects called by two or more readers were included as ground-truth annotations in each image. Each set increased the stringency for a human-segmented object to be included until the fifth and final set, in which each object in the ground truth was called by all five human readers. Cellpose2.0 was evaluated against each of these ground-truth sets.

#### 2.4.5 Statistical analysis and data visualization

The Mann–Whitney U-test^30^ with Bonferroni correction^31^ for multiplicity was used to evaluate the differences in performance in terms of the means of the Jaccard and Sørensen indices for all comparisons in the performance described above. All p-values are reported after correction for multiplicity. For testing the differences between the mean pairwise metrics and the multireader generalizations, p-values were corrected for three comparisons. When evaluating the performance of each human reader relative to the group, p-values were corrected for fifteen comparisons. Finally, when evaluating humans and computer algorithms relative to the group, p-values were corrected for 18 comparisons. When comparing the mean pairwise metrics to the multireader generalizations, we tested the alternative hypothesis that the sample means were different. When evaluating reader quality and the algorithms relative to the group of human readers, we tested the alternative hypothesis that the smaller group of readers had a higher sample mean. Noninferiority of the two DCNNs relative to the group of human readers was tested by calculating the lower bound of the 90% confidence interval of the difference in the multireader Jaccard index. The multireader Jaccard index, a measure of agreement across a group, can marginally increase when adding a reader. However, this increase does not mean that the new reader is better than the group, but that they are very similar to the group. If adding a new reader to the group causes a decrease in the multireader Jaccard index, this means that the reader is dissimilar to the original group. Therefore, noninferiority testing is appropriate for testing if the level of agreement does not decrease when adding a reader. A Kruskal–Wallis test,^32^ the nonparametric equivalent of ANOVA, was used to evaluate whether the performance of Cellpose2.0 varied with the stringency at which ground truth was defined with regard to consensus across human readers. Violin plots are used to visually compare the sample means while maintaining a visual display of the data distribution within each group.

## 3 Results

### 3.1 Interreader Agreement: Pairwise and Multireader Metrics

**Fig. 5:**
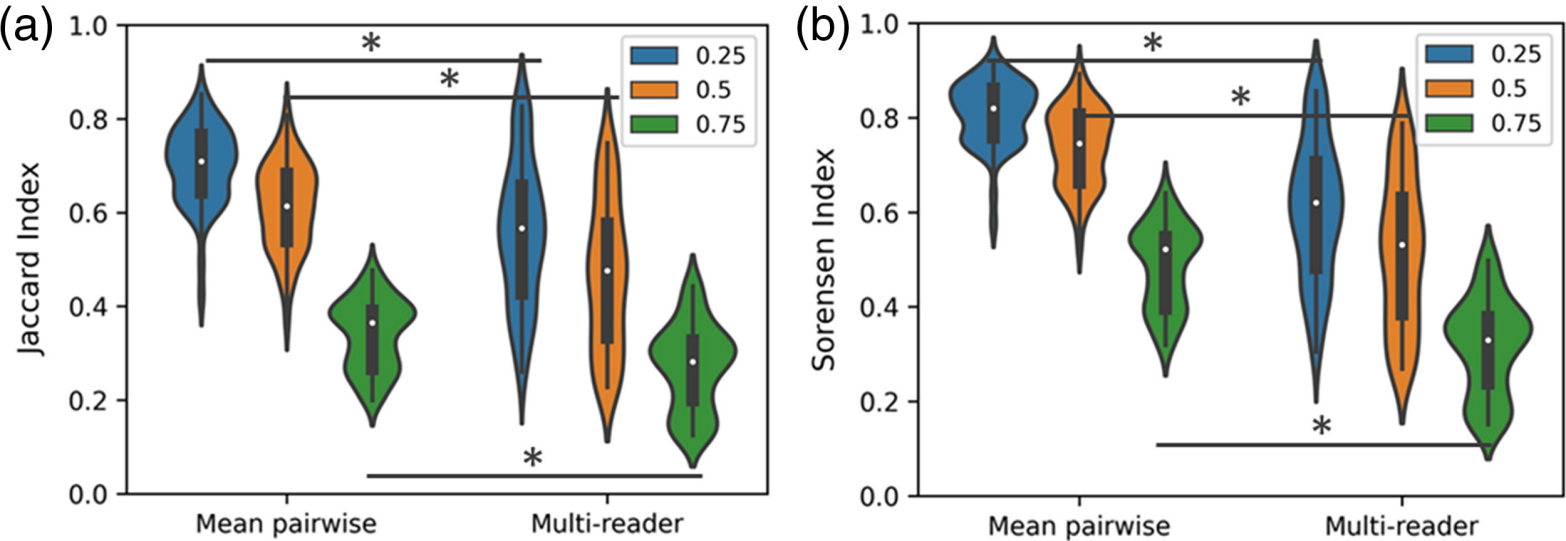
Violin plots of the Jaccard index (a) and Sørensen index (b) calculated as pairwise comparisons of readers and through the multireader generalization. Both metrics are compared across a range of IOU thresholds. At each threshold, a significant difference was found between the mean pairwise comparison and the multireader generalization. (* p<0.05, Mann–Whitney U-test with Bonferroni correction).

All images in the DAPI dataset were segmented by the five human readers. These segmentations were compared across readers in a pairwise fashion using the Jaccard and Sørensen indices and using the multireader generalizations of these metrics discussed above (Table 1 and Fig. 5). This resulted in each of the 50 images having 10 unique pairwise comparisons between readers. To compare overall agreement across readers, we show the distributions of two metrics: the mean of the unique pairwise comparisons per image and the multireader generalization calculated for each image. Both the Jaccard index [Fig. 5(a)] and Sørensen index [Fig. 5(b)] were different across all IOU thresholds when evaluating the mean pairwise comparison relative to the multireader generalization (p<0.05).

**Table 1:** Comparison of the mean of the unique pairwise comparisons across readers and the multireader generalization for each image in the dataset. Each metric is reported across a range of IOU thresholds, which are used to determine a prediction as a TP or an agreed-upon object. The mean and 95% confidence interval of the mean are reported for each metric.

	IOU threshold = 0.25		IOU threshold = 0.50		IOU threshold = 0.75	
	Jaccard index	Sørensen index	Jaccard index	Sørensen index	Jaccard index	Sørensen index
Mean pairwise comparison	0.69 [0.68:0.72]	0.81 [0.79:0.82]	0.61 [0.59:0.63]	0.74 [0.72:0.75]	0.34 [0.32:0.35]	0.48 [0.47:0.50]
Multireader generalization	0.55 [0.52:0.57]	0.60 [0.57:0.61]	0.46 [0.43:0.48]	0.51 [0.49:0.53]	0.27 [0.26:0.29]	0.32 [0.30:0.33]

### 3.2 Intrareader Agreement

Table 2 lists the Jaccard and Sørensen indices for three human readers at three different IOU thresholds. For this resegmentation task, agreement varied across readers, suggesting that some readers might be more consistent than others. For Secs. 3.2–3.4, reader IDs (Table 2, column 1) are consistent across all tables and figures.

**Table 2:** Evaluation metrics for comparing the performance of human readers across resegmenting cells. All metrics were calculated for a single reader segmenting each image twice, with a minimum of 4 weeks between segmentations. Reader IDs (column 1) are consistent for all downstream analyses. The mean and 95% confidence interval of each metric are reported for IOU thresholds of 0.25, 0.5, and 0.75.

Reader	Jaccard index (IOU ≥ 0.25)	Sørensen index (IOU ≥ 0.25)	Jaccard index (IOU ≥ 0.50)	Sørensen index (IOU ≥ 0.50)	Jaccard index (IOU ≥ 0.75)	Sørensen Index (IOU ≥ 0.75)
1	0.59 [0.54:0.63]	0.73 [0.69:0.77]	0.49 [0.41:0.51]	0.61 [0.56:0.66]	0.25 [0.21:0.28]	0.38 [0.33:0.43]
3	0.81 [0.76:0.85]	0.88 [0.84:0.92]	0.78 [0.74:0.83]	0.87 [0.83:0.91]	0.58 [0.54:0.63]	0.72 [0.68:0.76]
5	0.85 [0.83:0.88]	0.92 [0.91:0.93]	0.81 [0.79:0.84]	0.89 [0.88:0.91]	0.57 [0.53:0.62]	0.72 [0.68:0.75]

### 3.3 Reader Quality

**Fig. 6:**
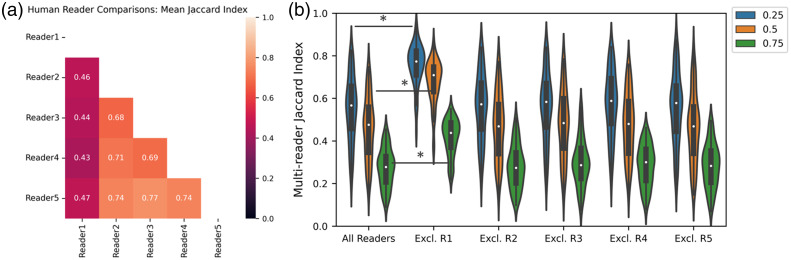
(a) Average Jaccard index across images for each pairwise combination of readers is shown for an IOU threshold of agreement of 0.5. (b) Each unique group of four readers was compared with the full group of five readers. Comparisons were performed at each IOU threshold. When excluding reader 1 (R1) from the group, the multireader Jaccard index significantly increased at every IOU threshold (p<0.0001).

The mean Jaccard index for each pairwise comparison of human readers shows that reader 1 agreed with all other readers at a lower rate than any other reader [Fig. 6(a)]. When reader 1 was removed from our pool of readers, the agreement between readers significantly increased [p<0.0001, Fig. 6(b)], suggesting that reader 1 was performing out of distribution relative to the other readers. When removing other readers from the pool, we failed to show a significant difference in the multireader Jaccard index relative to the full group. Interestingly, reader 1 was also the least consistent in the resegmentation task. This analysis demonstrates that the multireader generalization of the Jaccard index is capable of identifying when a reader is significantly different from a group of readers.

### 3.4 Human and Computer Agreement

**Fig. 7:**
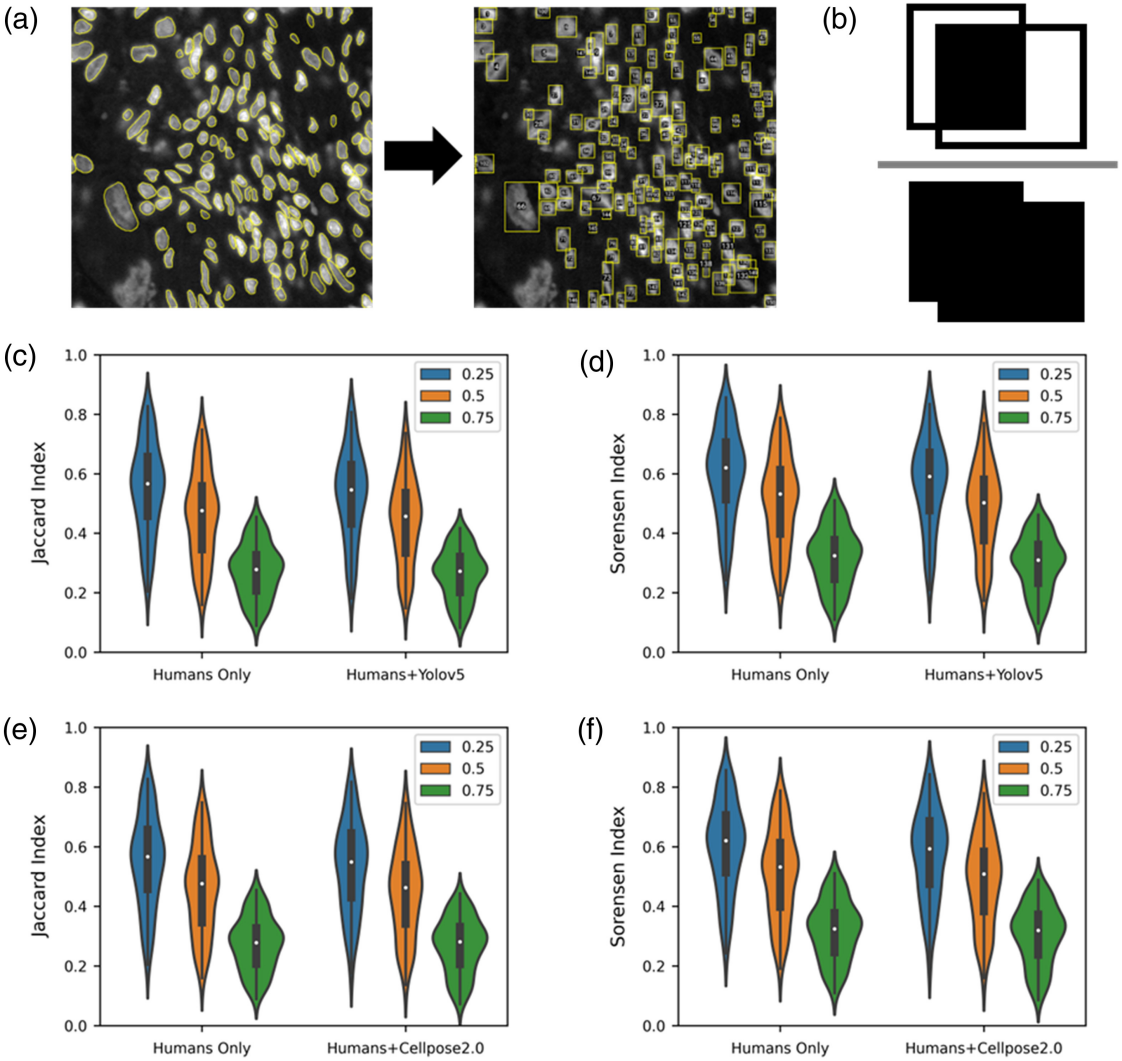
Two DCNNs (Yolov5 and Cellpose2.0) are compared with human readers for DAPI detection. (a) For comparisons with Yolov5, manual segmentations from human readers were converted to bounding boxes to compare the performance of human readers with Yolov5 in the task of calling cells. (b) A representation of bounding box IOU is shown. A violin plot comparing the distribution of the (c) multireader Jaccard index and (d) multireader Sørensen index for human readers only (left) and for human readers plus Yolov5 (right). A violin plot comparing the distribution of (e) multireader Jaccard index and (f) multireader Sørensen index for human readers only (left) and for human readers plus Cellpose2.0 (right).

In evaluating the algorithm performance, we first assessed whether Yolov5 performed similarly to a group of human readers in the task of object detection. Here, we discuss a “humans only” group and a “humans + Yolov5” group. For all proceeding analyses, this “humans + algorithm” group includes five human readers that independently annotated images and a sixth reader (algorithm) that also independently annotated the images. Yolov5 performs object detection, so all comparisons were calculated on cell bounding boxes extracted from the manual segmentations [Fig. 7(a)]. Bounding box IOU was used for defining agreement [Fig. 7(b)]. A cell was determined to be an agreement if the IOU of its bounding box with a bounding box from another reader was >0.25, 0.5, and 0.75. As expected, the degree of agreement decreased as the IOU threshold increased [Figs. 7(c) and 7(d), Table 3]. The difference in means between the human readers and the human readers plus Yolov5 failed to reach significance at any IOU threshold (p>0.05). The lower bounds of the 90% confidence interval for the difference in the multireader Jaccard index between the humans only group and the humans + Yolov5 group were −0.026, −0.019, and −0.014 for IOU thresholds of 0.25, 0.5, and 0.75, respectively, demonstrating noninferiority at all tested IOU thresholds.

**Table 3:** Mean and 95% confidence intervals of the multireader Jaccard and multireader Sørensen indices are reported for IOU thresholds 0.25, 0.5, and 0.75. Comparisons involving Yolov5 are computed on the bounding box around (Bbox), and comparisons with Cellpose2.0 are computed on the cell mask (seg). We failed to find significant differences between the five human readers and the five human readers + an algorithm (p>0.05).

	Multireader Jaccard index	Multireader Sørensen index
IOU threshold: 0.25		
Human readers only (Bbox)	0.55 [0.52:0.57]	0.60 [0.57:0.62]
Human readers + Yolov5 (Bbox)	0.53 [0.50:0.55]	0.57 [0.54:0.59]
Human readers only (seg)	0.55 [0.52:0.57]	0.60 [0.57:0.62]
Human readers + Cellpose2.0 (seg)	0.51 [0.49:0.56]	0.57 [0.55:0.60]
IOU threshold: 0.50		
Human readers only (Bbox)	0.46 [0.43:0.48]	0.51 [0.49:0.53]
Human readers + Yolov5 (Bbox)	0.44 [0.42:0.47]	0.48 [0.46:0.51]
Human readers only (seg)	0.46 [0.43:0.48]	0.51 [0.49:0.53]
Human readers + Cellpose2.0 (seg)	0.45 [0.42:0.47]	0.49 [0.46:0.51]
IOU threshold: 0.75		
Human readers only (Bbox)	0.27 [0.26:0.29]	0.32 [0.30:0.33]
Human readers + Yolov5 (Bbox)	0.26 [0.25:0.28]	0.30 [0.28:0.31]
Human readers only (seg)	0.27 [0.26:0.29]	0.32 [0.30:0.33]
Human readers + Cellpose2.0 (seg)	0.27 [0.25:0.28]	0.30 [0.29:0.32]

Although we compared agreement of bounding boxes for evaluating Yolov5 performance, we used the cell segmentations (while maintaining discrete objects) to compute IOU when comparing with Cellpose2.0 predictions [Table 3, Figs. 7(e) and 7(f)]. We failed to find a statistically significant difference between the group of human readers and the group of human readers plus Cellpose2.0 (p>0.05). The lower bounds of the 90% confidence intervals for the difference in the multireader Jaccard index at the three tested IOU thresholds were −0.022, −0.016, and −0.008. Therefore, Cellpose2.0 also cannot be shown to perform significantly worse than the group of human readers at any IOU threshold.

**Fig. 8:**
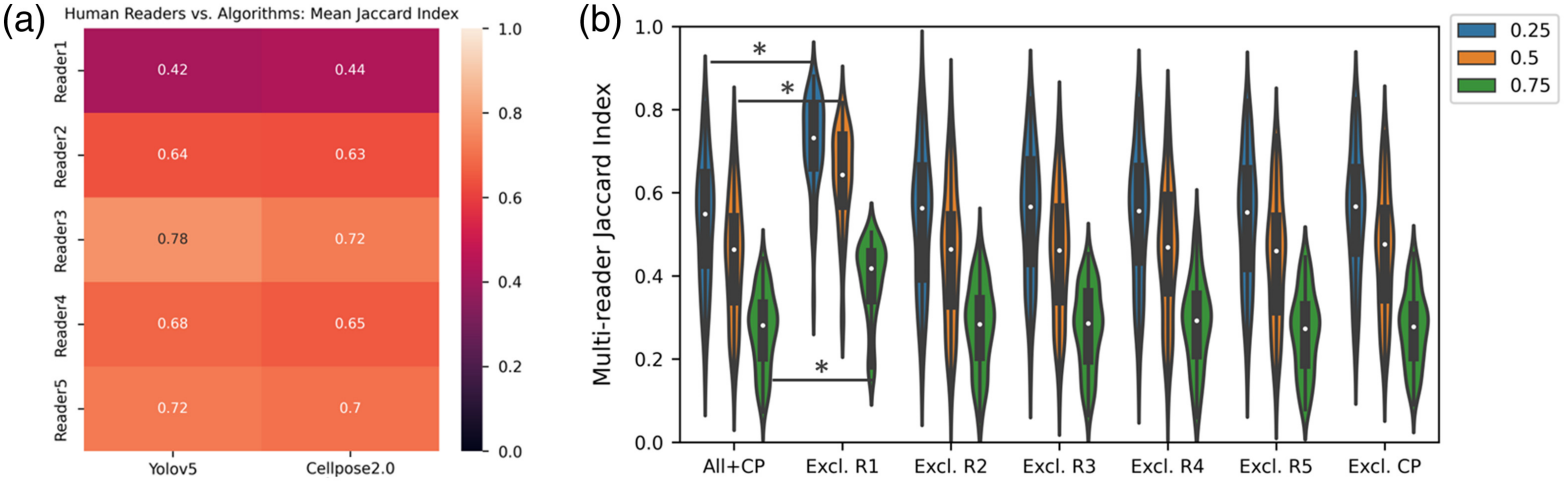
(a) Heatmap of the mean Jaccard index for each human reader compared with Yolov5 and Cellpose2.0 at an IOU threshold of 0.5. (b) Each unique group of five readers was compared with the full group of five human readers plus Cellpose2.0. Comparisons were performed at each IOU threshold. When excluding reader 1 (R1) from the group, the multireader Jaccard index increased at every IOU threshold relative to the full group of six readers (p<0.0001).

Yolov5 and Cellpose2.0 were next evaluated against each human reader [Fig. 8(a)]. Similar to the interreader studies with only human readers, reader 1 showed a lower agreement with each of the models relative to other human readers. Treating Yolov5 and Cellpose2.0 as readers, each reader was removed from the group, and the agreement across all readers was compared with the group without each reader. For both cases, removing the algorithm from the group failed to show an increase in agreement across readers [Fig. 8(b), data only shown for Cellpose2.0]. However, removing reader 1 from the group did show an increase in agreement across readers, indicating that Cellpose2.0 and Yolov5 agreed more with readers 2 to 5 than reader 1 agreed with either the group of human readers or the human readers plus an algorithm.

**Fig. 9:**
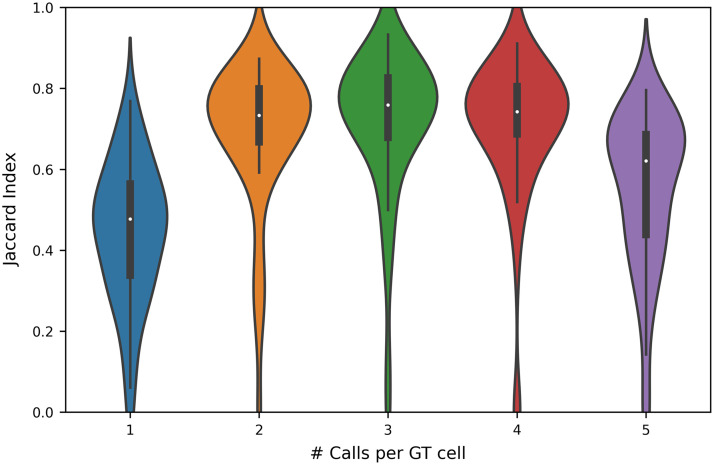
Cellpose2.0 evaluated against ground-truth sets selected by the number of manual annotations associated with each agreement from the set of five readers. A Kruskal–Wallis test shows that Cellpose2.0 performance varies when the stringency of the test set varies (p<0.0001).

Finally, Cellpose2.0 was evaluated against five test sets, which were defined by varying the level of agreement between human readers required for reader call to be included in the ground truth (Fig. 9). As expected, the performance of Cellpose2.0 varied with the number of calls per object required to define an object as ground truth (p<0.0001). Cellpose2.0 demonstrated the highest Jaccard index when compared with the test set, in which each ground-truth object was identified by a minimum of three readers (in this case, a majority).

## 4 Discussion

Human readers are notoriously inconsistent when defining ground truth for object-level computer vision tasks including object counting, object detection, and instance segmentation.^15^ Although cell detection, counting, and segmentation are subclinical tasks, acquiring accurate quantification of cells can provide valuable insight for both biomedical discovery and clinical decision making.^33^ However, this is not a task for human experts to perform in the clinic, so cell counting is a rare case in which we seek to fully remove the human element from image analysis. Currently, we still need manual annotations to verify the performance of cell detection and counting algorithms. We know that there is a large variance across human readers in these tasks,^34^^,^^35^ so consensus across readers would be helpful to optimizing algorithm training and test sets. However, it is nontrivial to quantify the agreement across multiple readers for such tasks. The multireader generalizations of the Jaccard and Sørensen indices presented in this work provide a way to evaluate reader agreement on object-level tasks. Furthermore, these metrics provide a singular, independent metric for defining reader agreement without averaging over pairwise agreements between readers. The resulting agreement values from these multireader metrics are significantly lower than pairwise means, but we believe that they better reflect the discrepancies within and between readers for object-level computer vision tasks.

We have demonstrated that these multireader metrics are valuable for identifying readers who perform differently from the consensus of other readers and for comparing the performance of computer algorithms (DCNNs in our application) with noisy reader labels. Increases and decreases in agreement can be measured when adding or removing readers (human or computer) from the group. If adding or removing a reader increases the average fraction of readers that call an object, these agreement metrics will increase. Similarly, if the mean fraction of readers that call an object decreases, these metrics will also decrease. Therefore, these multireader generalizations can help to determine whether adding a reader to the group—human or computer—will affect agreement among the reader pool. Rejecting a reader as inconsistent might prove to be controversial. In particular, if all readers are assumed to be equivalent, variability in annotations might simply be reflective of task difficulty.

We have also delineated methods for defining object-level consensus across several readers, allowing for rapid curating of ground truth based on how many readers called each object. We confirmed that the stringency with which a test set is defined from multiple readers affects the apparent algorithm performance as expected. It is important to note that if a singular set of ground truth is desired, a thorough analysis of the agreement between readers should be performed prior to selecting the number of calls that define a ground-truth object. Our analysis simply demonstrates that if object-level annotations are collected from multiple readers, the stringency with which you define your test set will affect the apparent performance of an algorithm. We prefer to compare the algorithm with the group of readers directly using the multireader metrics to determine whether an algorithm’s performance is within the distribution of human readers. Regardless of the method for curating manual annotations, the methods laid out in this work provide tools for understanding and characterizing the variability on manual annotations in object-level tasks.

DCNNs trained for object detection (Yolov5) and instance segmentation (Cellpose2.0) were compared with the group of human readers using multireader generalizations of the Jaccard and Sørensen indices. We found that the models performed comparably to the pool of human readers. The human readers in this study were novices in annotating cells in fluorescence microscopy images. However, we seek to show the applicability of these performance metrics rather than achieve an optimal ground-truth set. Here we evaluate algorithms on a single channel of fluorescence image data. However, instance segmentation of cell nuclei, although challenging, is among the easiest of tasks that require manual annotations in microscopy. Analyzing multiplex fluorescence images requires the definition of multiple cell classes based on the differential expression of the cells in various protein channels. Human readers’ performance is worse at multiclass object detection and instance segmentation tasks, which inherently limits the training of supervised algorithms for these tasks. However, being able to characterize the variability of manual annotations more fully could help to better curate training data for these tasks.

Although these generalizations provide a tool for describing multireader agreement on object detection and instance segmentation tasks, there are limitations to this work. We only demonstrate the multireader metrics for a single class of objects, but the multireader generalizations of the Jaccard and Sørensen indices can also be generalized to multiple classes of objects. In the multireader, multiclass generalizations, agreements and calls would be summed across classes prior to calculating the multireader Jaccard or Sørensen indices described above. This ensures that class imbalance does not skew the metric. We have not yet performed experiments to validate these multireader, multiclass metrics, partly because our reader pool is comprised of novice readers. In a preliminary study, agreement on multiclass instance segmentation of cells was very low between these readers.^34^ To further test these metrics, we will either need to train our current readers more extensively or recruit readers with more experience in reading fluorescence microscopy images.

## 5 Conclusions

We present multireader generalizations of the Jaccard and Sørensen indices to better characterize the agreement between multiple readers in object detection and instance segmentation tasks. Additionally, these metrics can compare the performance of a computer algorithm with noisy reader labels. We demonstrate that these metrics can detect when a reader is performing out of distribution relative to the rest of the reader pool. These multireader metrics provide a tool for understanding the agreement of readers on object-level tasks.

## Data Availability

All code associated with this work and sample annotations from three readers in this study can be found at https://github.com/durkeems13/multireader_metrics.
